# Extracellular vesicles for Alzheimer’s disease therapy: mechanisms, engineering strategies, and clinical translation

**DOI:** 10.20517/evcna.2026.43

**Published:** 2026-07-28

**Authors:** Ailin Wu, Yan Zeng, Yilin Huang, Legao Chen, Shunyuan Guo, Jianwei Wang, Xiaozhou Mou, Xiaoru Chang

**Affiliations:** ^1^College of Pharmacy, Zhejiang University of Technology, Hangzhou 310014, Zhejiang, China.; ^2^Center for Rehabilitation Medicine, Department of Neurology, Zhejiang Provincial People’s Hospital, Affiliated People’s Hospital, Hangzhou Medical College, Hangzhou 310014, Zhejiang, China.; ^3^Translational Medicine Center, Zhejiang Provincial People’s Hospital, Affiliated People’s Hospital, Hangzhou Medical College, Hangzhou 310014, Zhejiang, China.; ^4^General Surgery, Cancer Center, Department of Vascular Surgery, Zhejiang Provincial People’s Hospital, Affiliated People’s Hospital, Hangzhou Medical College, Hangzhou 310014, Zhejiang, China.

**Keywords:** Alzheimer’s disease, extracellular vesicles, MISEV2023, engineering, clinical translation, gut-brain axis, intranasal delivery

## Abstract

Alzheimer’s disease (AD), a progressive neurodegenerative disorder, remains a major global health challenge owing to its complex pathogenesis and the presence of the blood-brain barrier (BBB), which substantially limits the delivery of effective therapeutics to the brain. Extracellular vesicles (EVs), which exhibit favorable biocompatibility, low immunogenicity, and the potential to cross or bypass the BBB under specific conditions, have emerged as promising therapeutic agents and delivery platforms for AD. This review focuses on the therapeutic potential of EV-based interventions in AD and summarizes recent advances in EV-mediated modulation of AD-related pathological processes, including amyloid-β (Aβ) clearance, tau protein regulation, neuroinflammation suppression, oxidative stress attenuation, and synaptic repair. Although EV-based therapies offer notable advantages, such as targeted BBB penetration and reduced immunogenic responses, their clinical translation remains constrained by safety concerns, including off-target effects, dose-dependent toxicity, and potential disturbances in neuroplasticity. In addition, this review discusses EV engineering strategies aimed at regulating the gut-brain axis (GBA), enhancing brain targeting, and advancing clinical translation. EV-based therapeutic interventions should therefore be developed within a safety-oriented framework supported by rigorous short- and long-term toxicological evaluation. Overall, this review highlights the therapeutic promise of EVs for AD while underscoring the need for rational engineering, standardized characterization, and safety-centered translational strategies to ensure clinical feasibility.

## INTRODUCTION

Alzheimer’s disease (AD) is a common neurodegenerative disorder in older adults, characterized by progressive cognitive decline and behavioral disturbances associated with degeneration of the central nervous system (CNS)^[1,2]^. As the most common form of dementia, AD accounts for approximately 60%-70% of all dementia cases, and the global number of people living with dementia is projected to exceed 139 million by 2050^[3,4]^. This rapidly increasing disease burden poses substantial challenges to healthcare systems and the global economy. The hallmark neuropathological features of AD include extracellular amyloid-β (Aβ) plaques, intracellular neurofibrillary tangles (NFTs) composed of hyperphosphorylated tau, neuronal loss, synaptic degeneration, granulovacuolar degeneration, and gliosis^[5-7]^. The accumulation of pathogenic Aβ can activate microglia to trigger chronic neuroinflammatory responses, thereby exacerbating tau phosphorylation and neuronal injury^[8]^. These interconnected pathological events may form a self-reinforcing cycle that links amyloid pathology, tau pathology, neuroinflammation, and progressive neurodegeneration^[9]^. A major obstacle to the development of effective AD therapeutics is the presence of the blood-brain barrier (BBB). Although the BBB plays a key role in protecting the brain by restricting the entry of harmful substances from the bloodstream, it also limits the penetration of many therapeutic agents, including both small-molecule drugs and biologics^[10]^. Many AD drug candidates, especially Aβ-targeting antibodies, have poor BBB permeability due to their size, polarity, or stability. As a result, they fail to reach effective concentrations in the brain, which may partly explain their limited success in clinical trials despite promising early results.

Extracellular vesicles (EVs) are nanoscale lipid bilayer particles secreted by most cell types and have emerged as a promising platform for disease modulation and targeted drug delivery in AD^[11]^. Due to their favorable biocompatibility, low immunogenicity, and natural ability to cross the BBB, EVs offer clear advantages as delivery vehicles^[12]^. They can carry various bioactive molecules, including nucleic acids, proteins, lipids, and metabolites, directly to recipient cells, enabling precise regulation of disease-related processes^[13,14]^. To improve consistency in EV research, the International Society for Extracellular Vesicles (ISEV) introduced the minimal information for studies of extracellular vesicles (MISEV2023) guidelines, which standardize EV classification, isolation, characterization and reporting, thereby enhancing reproducibility and clinical translation^[15,16]^. Stem cell-derived EVs (SC-EVs), particularly those derived from mesenchymal stem cells (MSCs), neural stem cells (NSCs), and induced pluripotent stem cells (iPSCs), have shown multiple therapeutic effects in AD models. These include reducing Aβ production, limiting tau phosphorylation, promoting synaptic plasticity, enhancing neurogenesis, and suppressing neuroinflammation and oxidative stress^[17-20]^. Notably, these effects partly reflect the functions of their parent cells while avoiding risks such as immune rejection and tumor formation. Recent advances in EV engineering strategies, including surface functionalization, cargo loading, stimulus-responsive release, and improved delivery methods such as intranasal administration, have further increased their brain-targeting ability and therapeutic potential. In addition to mammalian EVs, plant-derived exosome-like nanovesicles (PELNVs) are gaining attention as scalable and orally administered delivery platforms. Their potential to regulate the gut-brain axis (GBA) may provide added benefits for CNS intervention. Following the MISEV2023 guidelines, this review uses “extracellular vesicles (EVs)” as a general term for cell-derived lipid bilayer particles. The term “EVs” is used consistently, while “exosomes” is applied only when specifically defined in the cited studies.

EVs can regulate multiple pathological processes and also serve as delivery platforms, making them a promising strategy for next-generation AD therapies. However, AD is not caused by a single factor; rather, it results from a complex, interconnected network of pathological events, including Aβ accumulation, tau pathology, neuroinflammation, oxidative stress, mitochondrial dysfunction, synaptic impairment, reduced neurogenesis, and GBA imbalance. Therefore, EVs should not be regarded simply as passive carriers. Instead, they act as mediators of intercellular communication that can either spread signals or help restore biological balance. These effects depend on their cellular source, molecular cargo, disease stage, and engineering design. This review examines EV mechanisms of action, engineering approaches, delivery strategies, manufacturing requirements, safety considerations, and clinical translation potential in AD treatment.

## EVS IN THE INTERCONNECTED PATHOLOGICAL NETWORK OF AD

AD is not driven by a single pathological process, but rather by a self-reinforcing network involving Aβ accumulation, tau pathology, neuroinflammation, oxidative stress, mitochondrial dysfunction, synaptic impairment, reduced neurogenesis, and GBA imbalance^[21,22]^. These processes are closely interconnected. Aβ accumulation can activate microglia and astrocytes, whereas persistent neuroinflammation can promote tau phosphorylation and synaptic damage^[23,24]^. Mitochondrial dysfunction increases oxidative stress, while redox imbalance further accelerates protein aggregation and neuronal degeneration^[25,26]^. Downstream changes, including synaptic loss, demyelination, cholinergic dysfunction, and impaired hippocampal neurogenesis, directly contribute to cognitive decline^[27-30]^. Within this network, EVs should not be viewed as simple carriers acting on single targets. Instead, they act as mediators of intercellular communication, transferring proteins, lipids, nucleic acids, inflammatory factors, mitochondrial regulators, and neurotrophic signals among neurons, glial cells, immune cells, and GBA-related pathways. Depending on their origin, cargo, and disease context, EVs can either spread pathological Aβ and tau species and inflammatory signals or support Aβ clearance, immune regulation, mitochondrial stability, and neural repair. Their therapeutic potential in AD derives from this ability to reshape the pathological network, shifting it away from a self-reinforcing degenerative state toward clearance, repair, and homeostasis.

### EVs as bidirectional regulators of the Aβ pathological cascade

Aβ pathology is not an isolated event but a dynamic cascade involving Aβ production, aggregation, extracellular deposition, clearance, and intercellular propagation^[31]^. EVs participate in multiple stages of this cascade. They may regulate amyloidogenic processing of amyloid precursor protein (APP) and Aβ generation, bind extracellular Aβ and alter its aggregation state, promote microglial uptake and enzymatic degradation, or conversely transport pathogenic Aβ species between cells and facilitate disease progression^[32]^. Therefore, EVs function as bidirectional regulators of Aβ homeostasis. Aβ peptides are generated through the sequential cleavage of APP by β- and γ-secretases, producing Aβ40 and the more aggregation-prone Aβ42^[33]^. During the early phase of the Aβ cascade, neuron-derived EVs may influence Aβ burden by regulating APP processing and intracellular Aβ metabolism, partly through membrane-associated enzymes such as endothelin-converting enzyme (ECE)^[34]^. Dysregulation of EV-associated enzymatic activity may shift APP processing toward increased Aβ generation and amyloid deposition^[32]^. From a therapeutic perspective, native or engineered EVs capable of suppressing amyloidogenic processing, enhancing Aβ-degrading activity, or delivering regulatory miRNAs or enzymes may reduce Aβ production during the early stage of AD pathology^[35]^. Following production, soluble Aβ species can progressively assemble into oligomers, protofibrils, fibrils, and plaques^[31]^. EVs may influence this aggregation process by binding extracellular Aβ through lipid membranes, surface proteins, or associated glycans^[35,36]^. This interaction may have dual consequences. In some contexts, EVs may sequester soluble Aβ and reduce the pool of freely diffusible toxic oligomers. At the same time, EV membranes may create local sites where Aβ accumulates and aggregates under pathological conditions. Therefore, EV-associated Aβ should not be considered uniformly beneficial or detrimental. Its biological effects depend on Aβ conformation, EV membrane composition, recipient cell type, and the local inflammatory environment.

EVs derived from neurons, microglia, or stem cells may facilitate Aβ removal by enhancing microglial uptake and modulating immune responses^[17,35,37]^. Therapeutic EVs, including mesenchymal stem cell-derived EVs (MSC-EVs), bone marrow mesenchymal stem cell-derived EVs (BMSC-EVs), adipose-derived mesenchymal stem cell-derived EVs (ADSC-EVs) and glia-derived EVs, have been reported to reduce Aβ burden and improve cognitive performance in AD models^[38-41]^. Mechanistically, EVs may convert freely diffusible and toxic extracellular Aβ species into vesicle-associated cargo that can be more efficiently recognized and cleared by microglia. EVs can also facilitate Aβ spread under pathological conditions. EVs released from diseased neurons can carry Aβ and tau to recipient cells and promote the propagation of toxic proteins across neural networks^[42]^. Their beneficial or harmful effects depend on cellular origin, donor-cell state, molecular cargo, disease stage, and the local disease microenvironment. EVs from healthy or therapeutically modified cells may enhance Aβ clearance and provide neuroprotection, whereas EVs from stressed neurons or disease-associated glial cells may transport pathogenic proteins and pro-inflammatory factors. EVs influence Aβ pathology in a context-dependent way by regulating its production, aggregation, clearance, and intercellular spread. For EV-based AD therapy, the main challenge is to develop strategies that reduce amyloidogenic APP processing and toxic Aβ oligomer formation, enhance microglial clearance, and limit the transfer of harmful Aβ species. EV-mediated regulation of Aβ pathology is shown in Figure 1A. Representative EV-based approaches for reducing AD pathology are summarized in Table 1.

**Figure 1:**
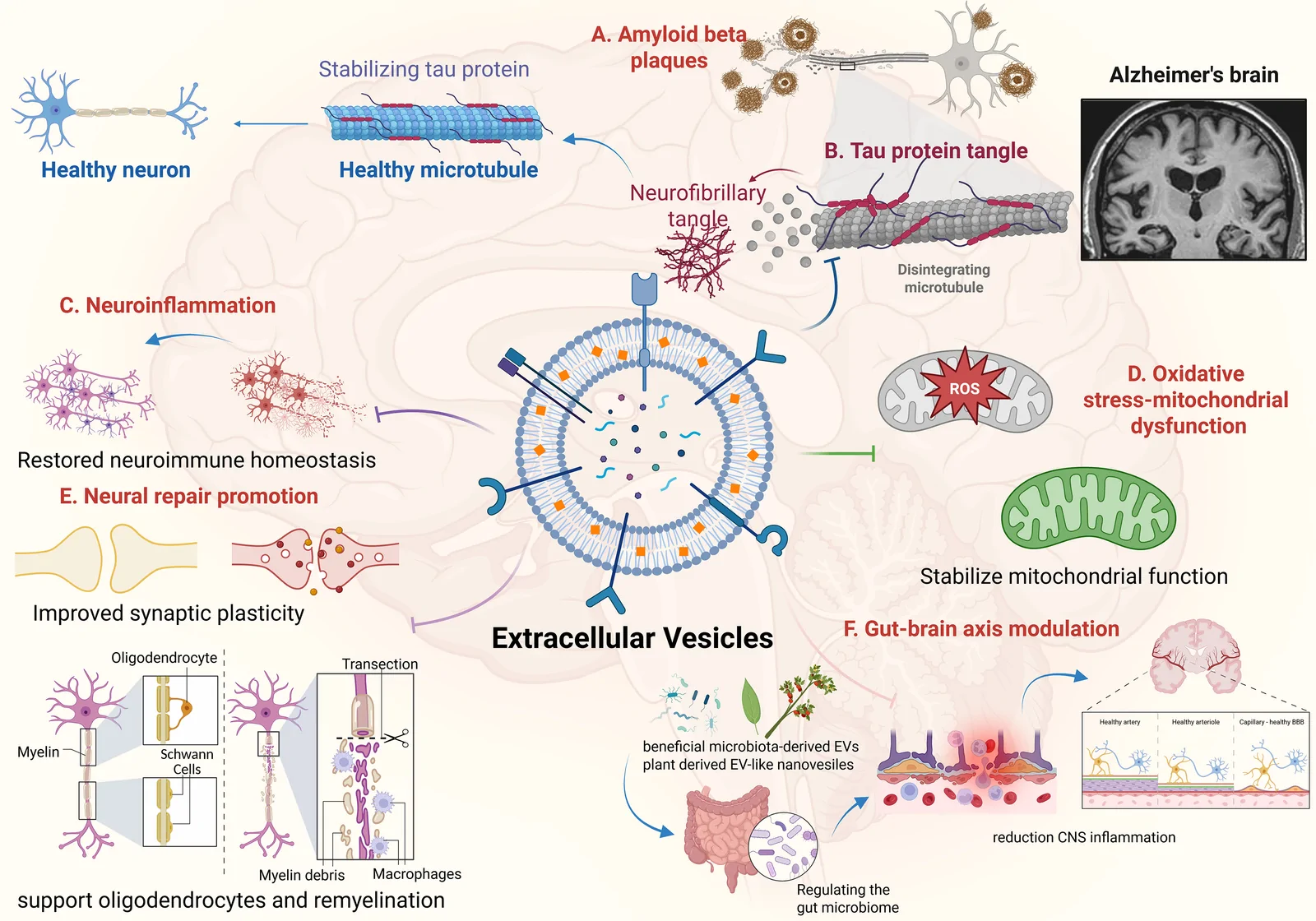
Mechanisms of EVs therapy in AD. Created in BioRender. (2026) https://BioRender.com/1uaabkm. AD: Alzheimer’s disease; BBB: blood-brain barrier; CNS: central nervous system; EVs: extracellular vesicles; ROS: reactive oxygen species.

**Table 1:** Therapeutic effects and mechanisms of EV-based in regulating AD pathology

**EV source/preparation**	**Therapeutic effects**	**Mechanistic pathways**	**Ref.**
BMSCs-EVs	Alleviated cognitive decline in AD-like mice	Improved BDNF-related neuropathology and neuronal support	[39]
ADSCs-EVs	Rescued memory deficits in APP/PS1 transgenic mice	Reduced brain-wide neural damage and markedly increased neurogenesis	[40]
Microglia-derived EVs	Reduced AD pathological burden	Synchronized macroautophagy and chaperone-mediated autophagy to enhance pathological protein clearance	[41]
NSCs-EVs	Improved learning and memory in AD mice	Reduced Aβ levels and tau phosphorylation, while restoring dendritic length and spine density	[17]
MSCs-EVs	Reduced chronic inflammation in 5 × FAD mice	Promoted Aβ clearance and preserved the tissue microenvironment required for neural recovery	[43]
hNSCs-EVs	Mitigated AD hallmarks	Reduced Aβ levels, inhibited microglial activation, conferred neuroprotection and prevented synaptic loss	[44]
Heat shock-induced NSC-EVs	Protected neurons from oxidative stress and Aβ-induced neurotoxicity	Stress-induced EV cargo remodeling enriched neuroprotective components and reduced Aβ levels	[45]
NSCs-EVs	Improved mitochondrial biogenesis and neuronal resilience	Activated PGC-1α/NRF1/TFAM signaling, improved mitochondrial biogenesis and restored abnormal protein distribution	[46]
M2 microglia-derived EVs	Attenuated neuronal impairment and mitochondrial dysfunction	Promoted PINK1/Parkin-mediated mitochondrial quality control	[47]
hUSSCs-EVs	Ameliorated spatial learning and memory deficits	Reduced Aβ accumulation and increased neuroplasticity protein expression	[48]
Lpc-EVs	Attenuated cognitive decline in Tg-APP/PS1 mice	Activated MeCP2/Sirt1 to enhance neurotrophic and Aβ-degrading pathways, improving Aβ pathology and cognition	[49]
Rhubarb-EVs	Restored cellular homeostasis	Protected against oxidative damage	[50]

In this table, the term “EVs” is used as the general term for extracellular vesicle preparations, including exosome-enriched fractions when the original studies described them as exosomes. AD: Alzheimer’s disease; ADSC-EVs: adipose-derived mesenchymal stem cell-derived extracellular vesicles; APP: amyloid precursor protein; Aβ: amyloid-beta; BDNF: brain-derived neurotrophic factor; BMSC-EVs: bone marrow mesenchymal stem cell-derived extracellular vesicles; EVs: extracellular vesicles; FAD: familial Alzheimer’s disease; hNSC-EVs: human neural stem cell-derived extracellular vesicles; hUSSC-EVs: 3D-cultured human unrestricted somatic stem cell-derived extracellular vesicles; Lpc-EVs: Lactobacillus paracasei-derived extracellular vesicles; MeCP2: methyl-CpG-binding protein 2; MSC-EVs: mesenchymal stem cell-derived extracellular vesicles; NRF1: nuclear respiratory factor 1; NSC-EVs: neural stem cell-derived extracellular vesicles; Parkin: Parkin RBR E3 ubiquitin protein ligase; PGC-1α: peroxisome proliferator-activated receptor gamma coactivator 1-alpha; PINK1: PTEN-induced kinase 1; PS1: presenilin 1; Rhubarb-EVs: rhubarb-derived extracellular vesicles; Sirt1: sirtuin 1; TFAM: mitochondrial transcription factor A; Tg: transgenic.

### EV-mediated regulation of tau pathology

In AD and other tauopathies, tau becomes abnormally hyperphosphorylated, which promotes the formation of NFTs. These tangles disrupt neuronal function and contribute to progressive neurodegeneration^[51,52]^. EVs may regulate tau homeostasis by participating in both intracellular and extracellular clearance pathways. Microglia-derived EVs, in particular, can sequester hyperphosphorylated tau and promote its degradation, thereby reducing intracellular tau accumulation^[53]^. The microglial receptor triggering receptor expressed on myeloid cells 2 (TREM2) has also emerged as a key regulator of tau clearance. Impaired TREM2 function exacerbates the accumulation of hyperphosphorylated tau, whereas normal TREM2 signaling facilitates tau trafficking through late endosomes, multivesicular bodies (MVBs), and EV-related pathways, thereby limiting pathological tau buildup^[54,55]^. The mechanisms underlying EV-mediated modulation of tau pathology are illustrated in Figure 1B.

In addition to regulating the phosphorylation of tau protein, EVs can also influence the aggregation and intercellular transmission of tau protein^[56]^. Pathological tau can be packaged in EVs from late endosomes/MVBs and released into the extracellular space, leading to aggregation in recipient neurons^[57]^. This EV-mediated transmission mechanism is considered to be an important way to promote the spread of tau pathology across synaptically connected neuronal networks. For example, in P301L tau transgenic mouse models, TREM2 deficiency accelerates the transfer of human tau from the medial entorhinal cortex to the dentate gyrus of the hippocampus, underscoring the role of microglial pathways in regulating tau dissemination^[58,59]^. Research has confirmed that disrupting the interaction between tau and EVs in microglia or inhibiting EV biogenesis/release can significantly reduce tau propagation in the brain. Therefore, therapeutic strategies that block EV-mediated tau transport by targeting cargo-loading mechanisms or modulating microglial EV release may provide a promising approach to delay the progression of tau pathology and alleviate neurodegenerative lesions in AD. These findings also highlight the need to strictly screen donor cells and accurately characterize EV cargo composition when developing EV-based therapeutic strategies.

### EV-mediated regulation of neuroinflammation and glial homeostasis

Neuroinflammation plays a key amplifying role in AD. It is associated with Aβ accumulation, tau pathology, mitochondrial dysfunction, and synaptic injury, and further aggravates these pathological processes^[60]^. Aβ deposition activates microglia and astrocytes, leading to cytokine release, oxidative stress, complement activation, and impaired synaptic homeostasis^[61]^. These inflammatory responses can further promote tau phosphorylation and mitochondrial damage, thereby forming a self-reinforcing pathological loop^[23]^. EVs participate in this loop by transferring immunomodulatory cargo between neurons, glial cells, and peripheral immune cells. MSC-EVs have demonstrated immunomodulatory effects by promoting the phenotypic transition of microglia from a pro-inflammatory M1-like state toward an anti-inflammatory and tissue-repair-associated M2-like state^[39,62]^. This shift reduces the production of pro-inflammatory mediators, enhances Aβ clearance, and mitigates pyroptosis^[16]^. In murine AD models, systemic or intranasal administration of MSC-EVs has been shown to reduce neuroinflammation and tau phosphorylation, and to improve spatial learning and memory^[63-65]^. In addition, NSC-EVs and induced neural stem cell-derived EVs (iNSC-EVs) effectively reduced Aβ and phosphorylated tau accumulation in the brains of five familial AD (5 × FAD) mice, inhibited neuronal injury, and attenuated microglia-driven neuroinflammation, suggesting that these EVs can ameliorate classical pathological features of AD^[17]^. Further modification or conditioning of MSCs may enhance the neuroprotective and immunoregulatory properties of their EVs^[43]^. These findings identify EV-mediated microglial reprogramming as a promising therapeutic strategy for controlling chronic neuroinflammation in AD. The mechanisms of EV-mediated regulation of neuroinflammation are summarized in Figure 1C. In addition to their effects on microglia, EVs can regulate the activation of astrocytes and the release of cytokines, thus helping to restore neuroimmune homeostasis and support neuronal survival^[42]^. Because astrocytes are involved in neurotransmitter recycling, metabolic regulation, synaptic support, and BBB maintenance, EV-mediated astrocyte modulation should be interpreted within the broader glial network rather than as an isolated anti-inflammatory effect.

### EV-mediated regulation of oxidative stress and mitochondrial dysfunction

Oxidative stress and mitochondrial dysfunction form a closely linked pathological axis in AD because they reinforce each other during disease progression [Figure 1D]^[66]^. Damaged mitochondria produce excessive reactive oxygen species (ROS), which further drive Aβ aggregation, tau phosphorylation, inflammatory responses, and synaptic damage^[67-69]^. EVs may help break this cycle by delivering antioxidant molecules, supporting mitochondrial quality control, and maintaining neuronal energy metabolism. SC-EVs retain key antioxidant and cytoprotective activities of their parent cells while exhibiting reduced immunogenicity, thereby providing a potentially safer and more controllable therapeutic alternative^[70-72]^. In particular, intranasally administered SC-EVs can cross the BBB and deliver antioxidant enzymes and regulatory molecules to injured brain regions^[73,74]^. Preclinical studies have shown that EV-based interventions can alleviate oxidative stress and neuroinflammation, promote tissue repair, and improve motor and cognitive function in AD-related models^[75]^. Under oxidative stress conditions, NSC-EVs may undergo adaptive cargo remodeling, enriching antioxidant and cytoprotective components that protect neurons against Aβ-induced oxidative injury and neurotoxicity^[44,45]^. EVs may also restore mitochondrial quality control. NSC-EVs have been reported to activate peroxisome proliferator-activated receptor gamma coactivator 1-alpha (PGC-1α)/nuclear respiratory factor 1 (NRF1)/mitochondrial transcription factor A (TFAM) signaling pathway, improve mitochondrial bioenergetics, and promote the redistribution of mitochondria to metabolically active neuronal regions^[46]^. In addition, EVs released by M2-polarized microglia can enhance PTEN-induced kinase 1 (PINK1)/Parkin-mediated mitophagy, thereby removing damaged mitochondria and restoring mitochondrial homeostasis^[47]^. Therefore, the therapeutic role of EVs in the oxidative stress-mitochondrial dysfunction axis is not limited to antioxidant delivery. Instead, EVs may simultaneously reduce ROS burden, restore mitochondrial dynamics, enhance mitophagy, and stabilize neuronal energy metabolism.

### EV-mediated neural repair: synaptic plasticity, myelination, cholinergic protection, and neurogenesis

Cognitive decline in AD is closely associated with the failure of multiple neural repair systems, including synaptic loss, dendritic spine degeneration, demyelination, cholinergic neuronal injury, and impaired hippocampal neurogenesis^[76-78]^. These repair deficits are not isolated events. They are downstream outcomes of an interconnected pathological network involving Aβ accumulation, tau pathology, neuroinflammation, and mitochondrial dysfunction. Aβ accumulation and tau pathology impair synaptic transmission, chronic neuroinflammation damages oligodendrocytes and cholinergic neurons^[79,80]^, and mitochondrial dysfunction reduces the energy supply required for synaptic maintenance, axonal conduction, and neuronal regeneration^[81,82]^. Therefore, EV-mediated neural repair can be understood as a functional restoration module that integrates synaptic preservation, myelin maintenance, cholinergic neuroprotection, and circuit remodeling. EVs may support synaptic repair by regulating synaptic protein expression, dendritic spine density, Wnt-related signaling, neurotrophic factor delivery, and mitochondrial function at synapses. EVs have been reported to modulate key proteins involved in synaptic architecture and neuronal communication. For example, EV-associated proline-rich protein 7 (PRR7) may interact with Wnt signaling pathways and regulate excitatory synaptic density, suggesting that EV-mediated signaling contributes to the maintenance of synaptic structure^[83]^. Engineered EVs carrying brain-derived neurotrophic factor (BDNF) or other neurotrophic cargo may cross the BBB, increase dendritic spine density, restore synaptic plasticity, and reduce neuroinflammatory injury^[84]^. In cellular and animal models of AD, MSC-EVs and 3D-cultured human unrestricted somatic stem cell (hUSSC)-EVs have been shown to reduce Aβ burden, restore memory-related gene expression, improve mitochondrial function, enhance synaptic activity, and improve cognitive performance^[48,85]^. Thus, EV-mediated synaptic repair may link upstream pathological modulation with downstream functional recovery [Figure 1E].

In addition to preserving synaptic integrity, EVs may support oligodendrocyte survival and myelin maintenance [Figure 1E]^[86]^. Myelin integrity is essential for axonal conduction, neural network synchrony, BBB stability, and neurovascular coupling^[87,88]^. In AD, oligodendrocytes are vulnerable to Aβ toxicity, inflammatory cytokines, oxidative stress, and mitochondrial impairment, which collectively contribute to progressive white matter damage and impaired neuronal communication^[89,90]^. EVs can deliver regulatory RNAs, proteins, and lipids that modulate oligodendrocyte differentiation, myelin protein expression and myelin repair^[91,92]^. For example, EV-associated cargo may regulate the expression of myelin-related proteins, whereas microglia-derived EVs have been reported to promote remyelination through the miR-615-5p/MYRF axis^[93]^. Because EV-mediated myelin repair is closely linked to neuroinflammation regulation and BBB maintenance, this mechanism should be interpreted as part of a broader neural repair process rather than as an isolated remyelination process.

EVs may also protect cholinergic neurons, which are particularly vulnerable during the transition from mild cognitive impairment to AD dementia^[94]^. Cholinergic dysfunction is closely associated with age-related memory impairment and is especially evident in early AD, when postsynaptic cholinergic responsiveness may remain relatively preserved^[29]^. Clinically approved acetylcholinesterase inhibitors (AChEIs), including donepezil, rivastigmine, and galantamine, enhance cholinergic neurotransmission and provide symptomatic benefit; however, they do not halt the progression of neurodegeneration^[95-97]^. EV-based strategies may complement cholinergic therapy by acting upstream of neurotransmitter release. In particular, MSC-EVs may attenuate inflammatory stress, mitochondrial dysfunction, and apoptotic signaling in vulnerable cholinergic neurons^[98-100]^. Hypoxia-preconditioned BMSC-EVs were reported to enhance neuroprotection by inhibiting NOD-like receptor family pyrin domain-containing 3 (NLRP3) inflammasome activation ^[101]^. Although these findings may provide a mechanistic reference for the development of extracellular vesicle-based therapeutic strategies for AD, further validation in AD-specific models is required.

EVs may promote hippocampal neurogenesis and circuit remodeling^[102]^. Impaired neurogenesis in the dentate gyrus contributes to defective memory formation and reduced cognitive flexibility in AD^[103]^. BMSC-EVs may enhance neurogenesis and neuronal plasticity through activation of the Zeb2/Axin2 axis under the coordinated regulation of SOX10, Wnt/β-catenin, and endothelin receptor type B (EDNRB) signaling pathways^[104]^. Similarly, induced NSC-EVs exhibit neuroprotective and pro-neurogenic properties comparable to those of native NSC-EVs, while BDNF-loaded NSC-EVs may improve NSC survival under oxidative stress, attenuate microglial overactivation, and promote neuronal differentiation^[105,106]^. *In vivo* studies further suggest that NSC-EVs can enhance adult hippocampal neurogenesis and contribute to functional recovery in neurological disease models^[107]^.

Taken together, EV-mediated neural repair may serve as a downstream functional endpoint for EV-based AD therapy. By preserving synapses, supporting myelin maintenance, protecting cholinergic neurons, and promoting hippocampal neurogenesis, EVs may help translate upstream regulation of Aβ pathology, tau pathology, neuroinflammation and mitochondrial dysfunction into cognitive benefit. Effective neural repair depends on the collaborative integration of neural circuits, not on the improvement of isolated processes. Future research should use comprehensive outcome measures, including synaptic density, white matter integrity, cholinergic neuron survival, hippocampal neurogenesis, electrophysiological activity, and cognitive performance, to evaluate EV-mediated neural repair effects.

### EV-mediated gut-brain communication in AD therapy

Accumulating evidence indicates that alterations in gut microbiota composition, commonly referred to as gut dysbiosis, play an important role in the pathogenesis of AD^[108]^. EVs may influence neurodegenerative pathology by regulating intestinal barrier integrity, systemic inflammation, BBB permeability, microglial activation, oxidative stress, and metabolic signaling [Figure 1F]^[109-111]^. Microbiota-derived EVs may act as double-edged mediators in this process. EVs released by pathogenic bacteria can carry pro-inflammatory molecules, thereby increasing systemic inflammation, disrupting BBB integrity, activating glial cells, and indirectly promoting Aβ accumulation or tau pathology^[112]^. In contrast, EVs derived from beneficial bacteria may carry anti-inflammatory and immunomodulatory signals that help restore gut barrier function, reduce peripheral inflammation, and attenuate neuroinflammatory responses^[49]^. Probiotic-derived EVs provide an EV-centered explanation for how gut-targeted interventions may influence AD-related pathology. Probiotic-derived EVs can be considered nanoscale mediators that transfer bacterial proteins, lipids, nucleic acids, and metabolites to host immune and epithelial cells. These signals may regulate systemic inflammation and microglial activation, thereby linking peripheral gut modulation with central neuroimmune responses.

PELNVs may have therapeutic potential for AD because they can be administered orally and may regulate the GBA through interactions with intestinal epithelial cells, immune cells, and gut microbiota^[50,113,114]^. PELNVs may indirectly modulate central pathology by reducing intestinal inflammation, reshaping microbial metabolism, and suppressing systemic inflammatory signals that contribute to microglial activation^[115]^. Therefore, their therapeutic value should be interpreted within the context of EV-mediated gut-brain communication, oral delivery, and AD-related neuroinflammation rather than being viewed merely as a general microbiota-targeted intervention. Overall, GBA-related EVs link peripheral inflammation, barrier function, metabolic signaling, and CNS pathology. This framework positions microbial EVs, probiotic-derived EVs, and PELNVs as interconnected mediators within a broader EV-based network that links Aβ/tau pathology, neuroinflammation, mitochondrial dysfunction, and neural repair.

## EV-BASED COMBINATION THERAPEUTIC STRATEGIES FOR AD

AD is a multifactorial neurodegenerative disease. Therefore, EV-based therapies are unlikely to fully address its pathological complexity through a single intervention. A more clinically realistic strategy may involve combining EV-based approaches with approved symptomatic treatments, disease-modifying antibodies, or lifestyle interventions. Such combination strategies could more precisely target the multiple pathological features of AD by synchronously modulating cognitive symptoms, amyloid pathology, neuroinflammation, mitochondrial dysfunction, and neural repair processes, thereby better aligning with the interconnected nature of the disease.

One feasible strategy is to combine EV-based therapy with AChEIs, such as donepezil, rivastigmine, and galantamine. As mentioned above, AChEIs mainly relieve symptoms by enhancing cholinergic neurotransmission^[116]^. EVs can exert non-cholinergic regulatory effects by reducing neuroinflammation, oxidative stress, mitochondrial dysfunction, neuronal apoptosis, and synaptic injury^[117]^. This mechanistic complementarity suggests that the combination of EVs and AChEIs can simultaneously target cognitive symptoms and underlying cellular damage in AD. Such combinations may provide symptomatic cholinergic support together with broader neuroprotective effects, especially for patients with early or mild-to-moderate AD. Further research is still needed to clarify the optimal dose, the timing of administration, the interaction between drugs and EV-based therapy, and long-term safety. Another promising strategy is to combine EV-based therapy with anti-Aβ antibodies. Anti-Aβ antibodies mainly reduce amyloid plaque burden, while EVs can regulate downstream pathological processes, including microglial activation, tau phosphorylation, mitochondrial dysfunction, oxidative stress, and synaptic degeneration^[41,44,46]^. Engineered EVs carrying anti-inflammatory molecules, miRNAs, siRNAs, neurotrophic factors, mitochondrial regulators, or small-molecule drugs can complement antibody-mediated amyloid clearance and provide broader neuroprotective effects^[118-120]^. Against this background, EVs may help shift the pathological network of AD from amyloid-driven inflammation and neuronal damage to immunomodulation, mitochondrial stabilization, and neural repair. However, such combination therapies need to be carefully evaluated, with particular attention to factors such as immune activation, vascular effects, amyloid-related imaging abnormalities, treatment timing, and patient selection.

EV-based therapy may also be combined with lifestyle interventions, such as dietary modification, physical exercise, and metabolic regulation. These interventions can influence systemic inflammation, gut microbiota, oxidative stress, insulin sensitivity, neurovascular function, and synaptic plasticity, all of which are closely associated with AD progression^[121]^. EVs may participate in these processes by mediating communication between the periphery and the CNS. For example, exercise- or diet-induced changes in circulating EV cargo may reflect or contribute to alterations in neuroinflammation, mitochondrial function, and neuronal repair^[122,123]^. Thus, EVs may also serve as biomarkers for monitoring physiological responses to lifestyle interventions. Combining EV-based therapies with exercise, dietary strategies, or metabolic interventions may provide a more personalized and multidimensional approach to AD management. EV-based combination therapy may better align with the multifactorial and interconnected nature of AD than single-target treatments. By integrating cholinergic symptom control, amyloid clearance, anti-inflammatory effects, mitochondrial support, and neural repair, such strategies may expand the therapeutic window of EV-based AD therapy. However, most of these approaches remain at the conceptual or preclinical stage. Further mechanistic, pharmacological, and clinical studies are needed to evaluate the therapeutic synergy of EV-based combination strategies and define optimal dosing regimens, treatment timing, safety profiles, biomarker responses, and the patient populations most likely to benefit.

## RECENT ADVANCES IN EV ISOLATION, PURIFICATION, QUALITY CONTROL AND SCALABLE MANUFACTURING

The translational potential of EVs as therapeutic carriers and bioactive agents for AD largely depends on the stability, purity, batch-to-batch consistency, and scalability of their production processes^[124]^. According to the MISEV2023 guidelines, EV studies should systematically report sample sources, preprocessing conditions, isolation and concentration methods, EV characterization parameters, and functional validation strategies to improve reproducibility and comparability across studies^[16]^. Evaluation of EVs for AD therapy should consider both their biological functions, including neuroprotection, anti-inflammatory effects, inhibition of Aβ deposition, and modulation of tau pathology, and the extent to which their isolation, purification, and quality control processes meet the standards required for clinical translation.

At present, the main EV isolation methods include differential ultracentrifugation, density gradient centrifugation, ultrafiltration, size-exclusion chromatography (SEC), immunoaffinity capture, and microfluidic technologies^[125]^. A comparison of commonly used EV isolation techniques is presented in Table 2. Differential ultracentrifugation is the most classical and widely used method and remains suitable for basic research. However, it is time-consuming, exhibits considerable variability in recovery efficiency, and may result in the co-precipitation of protein aggregates, lipoproteins, or other non-EV components^[126]^. Density gradient centrifugation can improve EV purity, but the procedure is technically complex, has limited yield, and is difficult to adapt to large-scale production^[124]^. SEC offers several advantages, including mild processing conditions and relatively good preservation of EV integrity and biological activity, and has therefore been increasingly applied to the purification of therapeutic EVs in recent years. However, its sample-processing capacity remains limited, and sample dilution may occur during separation^[127]^. In contrast, tangential flow filtration (TFF) provides better scalability, is suitable for processing large volumes of cell culture supernatants or biological fluid samples, and can be combined with SEC to balance concentration efficiency, purity, and preservation of biological activity^[128]^. In recent years, microfluidic platforms have received increasing attention as alternatives to traditional EV isolation methods. These systems integrate EV capture, purification, detection, and analysis into microdevices and offer several advantages, including low sample consumption, rapid processing, high automation, and precise separation of specific EV subpopulations^[129]^. Immunoaffinity capture enables the selective isolation of EVs based on specific surface markers or defined EV subpopulations. This method offers high specificity and is particularly useful for diagnostic applications, biomarker studies, and the enrichment of specific EV subgroups, such as brain-derived or neuron-derived EVs. However, immunoaffinity capture is limited by high cost and relatively low processing capacity, and the selection of specific markers may introduce bias by enriching only marker-positive EV populations^[130,131]^. In AD research, microfluidic technologies are valuable for the detection and analysis of brain-derived or neuron-derived EVs and downstream biomarker studies^[132]^. Nevertheless, several challenges remain, including limited standardization, variability in recovery efficiency due to differences in chip materials and antibody selection, interference from complex clinical samples, and difficulties in scaling up production^[133]^. Therefore, microfluidic technologies are currently more suitable for EV-based diagnostics and subpopulation analysis, whereas large-scale production of therapeutic EVs still requires integration with manufacturing processes that are easier to scale up.

**Table 2:** Comparison of EV isolation techniques: advantages, limitations, and application scenarios

**Technique**	**Advantages**	**Limitations**	**Application scenarios**	**Reference**
Ultracentrifugation	Classical and widely used; suitable for routine EV isolation in laboratory settings	Time-consuming; recovery rate and purity may vary; protein aggregates, lipoproteins, or other non-EV components may co-precipitate	Basic research and preclinical EV studies, especially when high-throughput or clinical-grade production is not required	[126]
Density gradient centrifugation	Provides higher EV purity by separating vesicles based on buoyant density	Complex workflow; limited yield; low throughput; difficult to adapt to large-scale production	High-purity EV preparation and mechanistic studies requiring reduced contamination	[124]
SEC	Gentle separation process; better preserves EV integrity and biological activity	Limited sample-processing capacity; may dilute EV preparations and require additional concentration steps	Functional studies and purification of therapeutic EVs where preservation of bioactivity is important	[127]
TFF	Scalable; suitable for processing large volumes of cell culture supernatants or body fluids; can be combined with SEC to balance concentration efficiency, purity, and bioactivity	Membrane fouling may occur; operating parameters require optimization to avoid EV loss or damage	GMP-compatible manufacturing, industrial production, and large-scale preparation of therapeutic EVs	[128]
Immunoaffinity capture	High specificity for selected EV markers or subpopulations	High cost; limited capacity; marker selection may bias the captured EV population	Diagnostics, biomarker studies, and isolation of specific EV subgroups such as brain-derived or neuron-derived EVs	[130,131]
Microfluidic technologies	Requires low sample volume; rapid and highly integrated; can combine EV capture, purification, detection, and analysis; suitable for precise EV subpopulation isolation	Insufficient standardization; chip materials and antibody selection may affect recovery; clinical samples are complex; industrial scale-up remains difficult	AD diagnostics, rapid detection, biomarker analysis, and precise isolation of brain- or neuron-derived EVs	[129]

AD: Alzheimer’s disease; EVs: extracellular vesicles; GMP: good manufacturing practice; SEC: size-exclusion chromatography; TFF: tangential flow filtration.

Quality control of therapeutic EVs is a core component of their clinical translation. An ideal EV formulation should be evaluated across multiple dimensions, including identity confirmation, purity, safety, potency, and stability. Common characterization methods include nanoparticle tracking analysis (NTA) or tunable resistive pulse sensing (TRPS) for assessing particle size distribution and concentration; transmission electron microscopy (TEM) or cryo-electron microscopy (cryo-EM) for observing vesicle morphology; and Western blotting, flow cytometry, or enzyme-linked immunosorbent assay (ELISA) to detect EV-enriched markers such as CD9, CD63, CD81, TSG101, and ALIX^[134-136]^. In parallel, non-EV contaminants or negative markers should also be assessed, together with potential contamination by free proteins or nucleic acids. For engineered or drug-loaded EVs, additional evaluations are required, including drug or nucleic acid loading efficiency, release kinetics, targeting capability, residual transfection reagents, residual host-cell DNA, sterility, mycoplasma contamination, and endotoxin levels^[124]^. In the context of AD therapy, EV quality control should also be integrated with disease-relevant functional assays. For example, the potency of EV formulations can be evaluated using assays related to Aβ aggregation or clearance, tau phosphorylation, microglial inflammatory responses, oxidative stress, neuronal survival, synaptic protein expression, and BBB penetration. Compared with assessments based solely on particle size, concentration, and surface markers, potency assays directly linked to AD-related pathological mechanisms are more informative for determining whether different EV batches retain consistent therapeutic activity. This consideration is important for MSC-EVs, NSC-EVs, engineered EVs, and PELNVs alike.

Scalable manufacturing is a key issue that must be addressed before EV-based therapies can advance toward clinical application. Current promising strategies include the use of serum-free or xeno-free culture systems, establishment of standardized donor cell banks, the adoption of 3D culture systems or bioreactors to increase EV yield, and downstream concentration and purification using TFF, SEC, or chromatography-based platforms^[137]^. For clinical-grade EV products, closed manufacturing processes under good manufacturing practice (GMP) conditions must also be established, with clearly defined control of raw materials, batch production records, release criteria, storage conditions, and transportation stability. Recent studies have emphasized that EV drug development requires not only demonstration of biological function but also the establishment of traceable, reproducible, and verifiable manufacturing and quality control systems^[124]^. Overall, EV isolation and purification technologies are gradually shifting from traditional laboratory-scale ultracentrifugation methods toward TFF, SEC, microfluidic systems, automation, and GMP-compatible platforms. For AD therapy, the key future challenge is not only to demonstrate the neuroprotective effects of EVs but also to establish a complete technical pipeline covering cell source, culture conditions, isolation and purification, quality control, potency validation, and scalable manufacturing. Only after the identity, purity, safety, stability, and therapeutic potency of EV formulations have been systematically validated can EV-based AD therapy progress from experimental research toward clinical translation.

In addition to their therapeutic potential, EVs are increasingly recognized as non-invasive diagnostic and early-stage biomarkers for AD. EVs isolated from biofluids such as blood, cerebrospinal fluid (CSF), saliva, or urine may carry disease-relevant cargo^[32]^. Because EVs partly reflect the molecular status of their parental cells, neuron-derived or brain-enriched EV subpopulations may provide valuable information for early diagnosis, disease staging, and pathological monitoring. EV-based biomarkers may also facilitate patient stratification, treatment response monitoring, target engagement assessment, and the identification of individuals most likely to benefit from EV-based or antibody-based therapies. Therefore, EV diagnostics may complement EV therapeutics and support a more personalized translational framework for AD management.

## HIERARCHICAL ENGINEERING STRATEGIES FOR EV-BASED AD THERAPY

Engineering strategies are central to improving the therapeutic performance of EVs in AD, as native EVs often exhibit insufficient brain targeting, heterogeneous cargo composition, variable therapeutic potency, and limited delivery efficiency^[138]^. However, EV engineering should not be regarded as a single technical category. Instead, different strategies act at distinct stages of EV development, including donor-cell manipulation, surface modification, therapeutic cargo loading, hybrid or biomimetic platform construction, stimuli-responsive release, and delivery-route optimization. Therefore, a hierarchical framework is needed to distinguish engineering methods, representative platforms, advantages, limitations, and translational considerations. In this review, EV engineering strategies for AD therapy are organized into six major levels: source-cell engineering, surface engineering, cargo engineering, hybrid or biomimetic EV platforms, stimuli-responsive EV systems, and delivery-route optimization. Source-cell engineering determines the intrinsic molecular cargo and biological activity of EVs. Surface engineering improves BBB penetration, brain targeting, cellular uptake, and circulation stability. Cargo engineering introduces therapeutic molecules that regulate Aβ pathology, tau pathology, neuroinflammation, oxidative stress, mitochondrial dysfunction, and neural repair. Hybrid or biomimetic systems combine EVs with synthetic platforms or cell membrane-based systems to enhance stability and targeting specificity. Stimuli-responsive systems allow controlled release of cargo at specific sites or times. Optimizing delivery routes can affect CNS exposure, systemic distribution, patient adherence, and the feasibility of clinical translation.

### Source-cell engineering and preconditioning

Source-cell engineering represents the upstream link of EV modification, because the type of donor cell and culture conditions largely determine the cargo composition, surface molecules, and biological functions of EVs^[139]^. EVs from MSCs, NSCs, iPSCs, microglia, dendritic cells, or plant sources can differ substantially in their protein, RNA, lipid, and metabolite profiles. In AD therapy, source-cell engineering is used to enrich EVs with neuroprotective, anti-inflammatory, antioxidant, mitochondria-supporting, or pro-repair cargo before isolation^[140]^. Common approaches include hypoxic preconditioning, inflammatory cytokine priming, genetic modification of donor cells, and disease-relevant environmental conditioning. For example, hypoxia-preconditioned MSC-EVs may enhance neuroprotective effects by increasing anti-inflammatory activity^[141]^. Genetic engineering of donor cells can promote the expression of targeting ligands, therapeutic RNAs, neurotrophic factors, or enzymes before EV release. Compared with post-isolation modification, source-cell engineering may better preserve EV membrane integrity and generate more biologically integrated cargo. However, this strategy also introduces variability related to donor-cell passage, culture medium composition, priming intensity, and batch-to-batch consistency. Therefore, source-cell engineering should be combined with strict donor-cell screening, standardized culture conditions, and comprehensive cargo profiling. Engineered vesicles for mitigating AD pathology are summarized in Table 3 and Figure 2.

**Figure 2:**
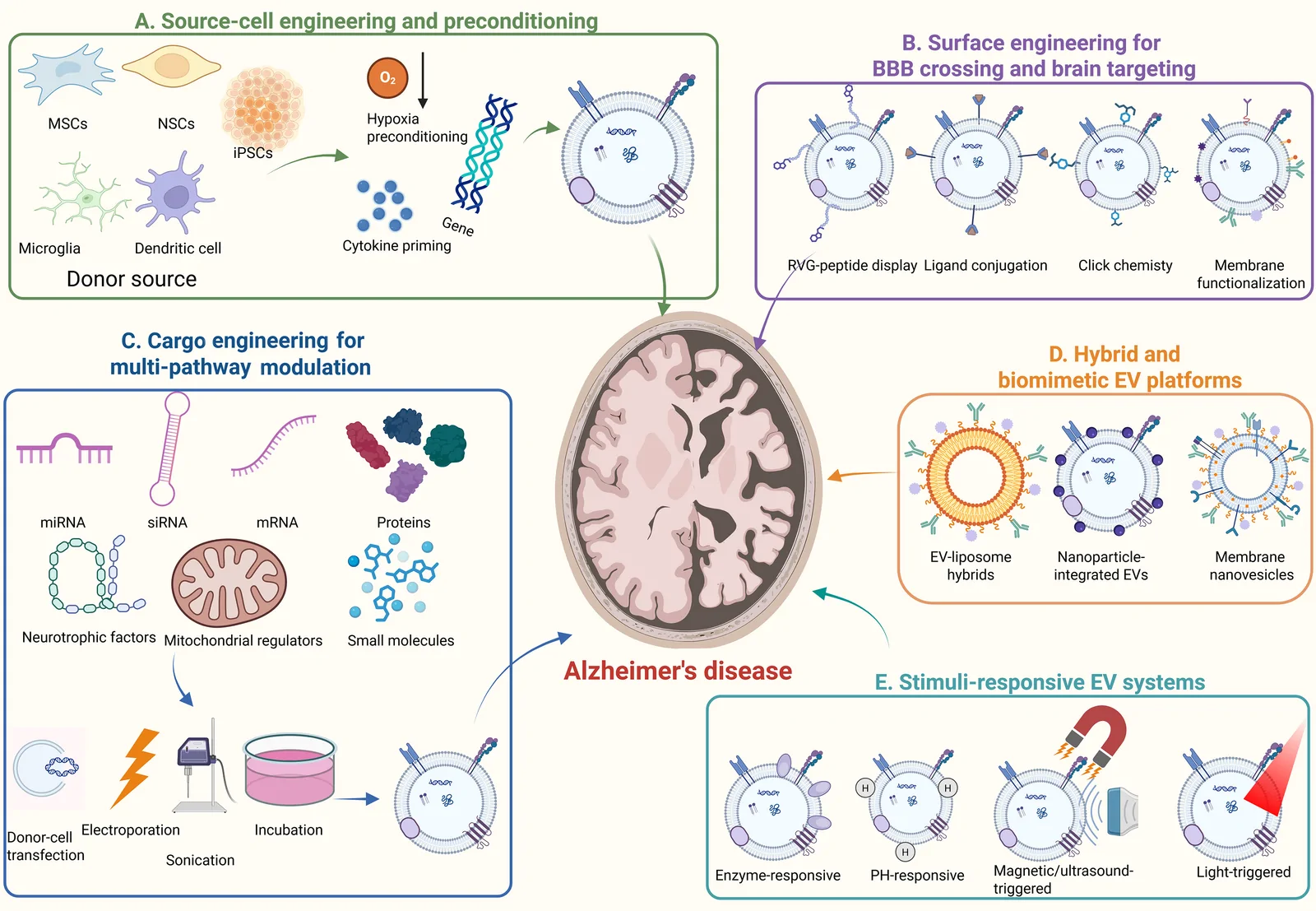
Engineering strategies of EVs for AD therapy. Created in BioRender. (2026) https://BioRender.com/rvrshvz. AD: Alzheimer’s disease; BBB: blood-brain barrier; EVs: extracellular vesicles; iPSCs: induced pluripotent stem cells; miRNA: microRNA; mRNA: messenger RNA; MSCs: mesenchymal stem cells; NSCs: neural stem cells; O_2_: oxygen; pH: potential of hydrogen; RVG: rabies virus glycoprotein; siRNA: small interfering RNA.

**Table 3:** Engineered EV strategies for AD therapy

**Engineering strategy**	**EV source/platform**	**Cargo/Modification**	**Therapeutic effects**	**Mechanistic pathways**	**Ref.**
Surface engineering	RVG-modified MSC-derived EVs	RVG peptide displayed on EV surface	Rescued memory deficits in an AD mouse model	Enhanced brain targeting and regulated inflammatory responses	[118]
Multi-target cargo/surface engineering	MP@Cur-MExo/engineered activated-neutrophil EVs	Curcumin plus multifunctional membrane components	Improved mitochondrial health and reduced AD-related pathological injury	Coordinated regulation of mitochondrial protection, Aβ-related injury, and inflammation	[119]
Donor-cell genetic engineering and drug loading	Fe65-EXO	Loaded with Corynoxine-B	Ameliorated cognition and AD pathology	Improved mitochondrial autophagy and neuronal homeostasis	[142]
Small-molecule cargo engineering	Curcumin-primed EVs	Curcumin	Improved cognitive function and reduced tau hyperphosphorylation	Inhibited tau hyperphosphorylation through the AKT/GSK-3β pathway	[143]
Small-molecule cargo engineering	Quercetin-loaded EVs	Quercetin	Improved cognition and reduced tau-related neurofibrillary pathology	Inhibited phosphorylated-tau-mediated neurofibrillary tangles	[144]
Small-molecule cargo engineering	Berberine/palmatine-EVs	Berberine and palmatine	Targeted neuroinflammation in AD	Modulated microglial inflammatory responses	[145]
Hybrid EV engineering	Biomimetic nanovesicles	ROS-responsive biomimetic EV-liposome hybrid nanovesicles	Improved cognitive impairment in APP/PS1 mice	Modulated microglia, reduced Aβ, and prevented neuroinflammation	[146]
Hybrid EV engineering	RPDA@Rb-A hybrid EVs	Hybridizing BMEC/macrophage exosomal membranes with PDA nanoparticles, resveratrol, and Aβ-targeting aptamers	Enhanced BBB penetration and alleviated Aβ pathology, neuroinflammation, and memory deficits	Acted as Aβ nanoscavengers and inflammatory modulators	[75]
Hybrid EV engineering	Engineered microglia-derived nanovesicles (AR@ENV)	Codelivery of AR7 and rapamycin	Rescued cognitive deficits in two AD mouse models	Enhanced toxic aggregate clearance, restored proteostasis, and provided robust neuroprotection	[41]
Light-triggered EV engineering	MAPLEX-like engineered EVs	Fusing them with photocleavable protein (mMaple3)	Improved cognition	Decreased Aβ levels	[147]
Enzyme-responsive EV hydrogel engineering	MSC-EV protease-triggered hydrogel system	Self-stimulated peptide hydrogel activated by EV membrane proteases	Rescued memory loss	Improved cognition, restored dendritic spine density, and promoted neurogenesis	[148]

AD: Alzheimer’s disease; AKT: protein kinase B; AR@ENV: AR7/rapamycin-loaded engineered microglia-derived nanovesicles; Aβ: amyloid-β; BBB: blood-brain barrier; BMECs: brain microvascular endothelial cells; CMA: chaperone-mediated autophagy; EVs: extracellular vesicles; Fe65-EXO: Fe65-engineered HT22 hippocampal neuron-derived extracellular vesicles; GSK-3β: glycogen synthase kinase-3β; HT22: HT22 hippocampal neuronal cell line; MAPLEX: magnetic-assisted phototherapy for light-triggered extracellular vesicle release; MP@Cur-MExo: curcumin-loaded engineered activated neutrophil-derived extracellular vesicles; MSC-EVs: mesenchymal stem cell-derived extracellular vesicles; PDA: polydopamine; PS1: presenilin 1; Res: resveratrol; ROS: reactive oxygen species; RPDA@Rb-A: resveratrol- and Aβ-targeting aptamer-modified engineered extracellular vesicles; RVG: rabies virus glycoprotein.

### Surface engineering for BBB crossing and brain targeting

Surface engineering modifies the outer membrane of EVs to improve biodistribution, BBB penetration, and cellular targeting^[149]^. This level of engineering is especially important for AD therapy because efficient delivery to the disease-relevant brain regions and cell types, including the cortex, hippocampus, microglia, astrocytes, and neurons, remains a major translational challenge. Current surface-engineering methods include genetic display of targeting peptides, covalent ligand conjugation, click chemistry, metabolic glycan labeling, enzymatic coupling, and affinity-based ligand attachment. Among these approaches, rabies virus glycoprotein-derived peptide (RVG peptide) modification is one of the most widely discussed and extensively investigated brain-targeting strategies. RVG-modified EVs can facilitate receptor-mediated transcytosis across the BBB and enhance delivery to AD-relevant brain regions, such as the cortex and hippocampus^[118]^. Multi-targeted EV platforms, such as MP@Cur-MExo, have shown the ability to preserve mitochondrial function, inhibit Aβ production and aggregation, and reduce neuroinflammation in AD models^[119]^. Other targeting ligands, antibodies, or peptides may also be used to improve neuronal or microglial uptake. A polydopamine (PDA)-functionalized M2-EV platform modified with RVG29 and a superparamagnetic iron oxide (SPIO)-based imaging probe enabled targeted brain delivery and real-time visualization in a mouse stroke model, reducing neuronal apoptosis and promoting neurological recovery^[150]^. Although this evidence comes from a stroke model rather than AD, it illustrates how surface engineering can integrate brain targeting and imaging-guided EV tracking. Surface lipid regulation and membrane protein engineering may also enhance circulation stability and cell-specific interactions. The main advantage of surface engineering is improved targeting specificity and delivery efficiency. However, surface modification may alter EV immunogenicity, clearance kinetics, receptor interactions, and organ accumulation. Therefore, surface-engineered EVs should be compared with unmodified EVs in terms of biodistribution, brain accumulation, immune responses, and repeated-dose safety studies.

### Cargo engineering for multi-pathway modulation

Cargo engineering defines the therapeutic mechanism of engineered EVs. EVs can be loaded with miRNAs, siRNAs, mRNAs, proteins, enzymes, neurotrophic factors, mitochondrial regulators, anti-inflammatory molecules, or small-molecule drugs^[151]^. These cargoes can be designed to target multiple AD-related pathological processes, including Aβ production and clearance, tau phosphorylation, neuroinflammation, oxidative stress, mitochondrial dysfunction, synaptic impairment, and neural repair. Donor-cell transfection or genetic modification can enrich EVs with specific RNAs or proteins during EV biogenesis^[152]^. Post-isolation loading approaches include passive incubation, electroporation, sonication, extrusion, freeze-thaw cycling, and chemical conjugation^[124]^. For example, RVG-modified dendritic cell-derived EVs loaded with BACE1 siRNA represent a dual-engineering strategy that combines brain-targeted surface modification with nucleic acid delivery for Aβ reduction^[120]^. EVs carrying mitochondrial autophagy regulators, such as Corynoxine-B, may improve mitochondrial quality control, whereas SHP2-overexpressing MSC-EVs may reduce neuroinflammation and restore mitochondrial function^[142]^. Small-molecule-loaded EVs, including EVs associated with curcumin, quercetin, berberine, or palmatine, have also shown potential for reducing Aβ or tau-related pathology in preclinical models^[143-145]^. The main advantage of cargo engineering is that it enables mechanism-specific and multi-target regulation. However, cargo loading efficiency, cargo stability, release kinetics, off-target gene regulation, innate immune activation, and dose-dependent toxicity remain major challenges. In addition, loading methods such as electroporation, sonication, or extrusion may damage EV membrane integrity or alter surface proteins. Therefore, cargo-engineered EVs should be systematically evaluated for loading efficiency, EV integrity, cargo release, potency, off-target effects, and long-term safety.

### Hybrid and biomimetic EV platforms

Hybrid or biomimetic EV platforms represent a more complex level of EV engineering, in which EVs are combined with liposomes, nanoparticles, cell membranes, magnetic materials, or other synthetic components. These platforms aim to integrate the biocompatibility and natural cell-communication properties of EVs with the controllability, stability, or loading capacity of synthetic nanocarriers^[153-155]^. In AD therapy, hybrid EV platforms may improve drug loading, enhance BBB penetration, prolong circulation time, or enable multifunctional targeting. For example, EV-liposome hybrids or biomimetic nanoparticles can be designed to regulate microglial activity, Aβ metabolism, or mitochondrial function^[146]^. RPDA@Rb-A, a PDA/Res-loaded hybrid EV platform with chol-Apt40-mediated Aβ targeting, enhanced BBB penetration and alleviated Aβ pathology, neuroinflammation, and memory deficits in AD mice^[75]^. AR@ENV is a microglia-derived nanovesicle that co-delivers AR7 and rapamycin to restore impaired macroautophagy and chaperone-mediated autophagy (CMA) in AD. Built using the Microglia-Liposome Fusion Extrusion (MiLi-FE) method, this platform can cross the BBB and target damaged neurons through microglial membrane-derived properties^[41]^. Multi-targeted EV systems, such as MP@Cur-MExo, have also shown the ability to preserve mitochondrial function, inhibit Aβ production and aggregation, and reduce neuroinflammation in AD models^[119]^. The main advantage of hybrid platforms is their functional flexibility. However, their increased structural complexity may reduce reproducibility and complicate safety evaluation. Synthetic components, hybrid membranes, magnetic particles, or chemical linkers may alter metabolic fate, immunogenicity, tissue retention, and long-term biocompatibility. Therefore, hybrid EV systems require more rigorous characterization than native EVs, including component identity, assembly stability, particle heterogeneity, degradation behavior, and comparative toxicity.

### Stimuli-responsive EV systems

Stimuli-responsive EV systems are designed to provide spatial and temporal control over cargo release. These systems respond to endogenous or exogenous triggers, such as pH changes, magnetic fields, light, enzymes, ultrasound, or disease-specific microenvironmental signals^[156]^. In AD therapy, stimuli-responsive systems may increase local drug exposure in pathological brain regions while reducing systemic exposure and off-target effects^[157]^. For example, pH-responsive EVs can preferentially release cargo in acidic or inflamed microenvironments^[158]^. Light-triggered systems, such as MAPLEX platforms based on light-responsive proteins, enable on-demand cargo release at defined sites and times^[147]^. An enzyme-responsive platform using MSC-EV membrane proteases, including FAP and dipeptidyl peptidase-4 (DPP4), as triggers for self-assembling peptide hydrogels improved cognition, restored dendritic spine density, promoted neurogenesis, and showed favorable safety profiles^[148]^. These strategies may expand the therapeutic window of EV-based interventions by improving release precision. However, their translational complexity remains high. Disease-stage heterogeneity may lead to variable pH profiles; magnetic components require long-term evaluation of tissue retention and biocompatibility; and repeated light exposure or photoresponsive proteins may introduce immune or tissue-compatibility concerns. Therefore, stimuli-responsive EV platforms should be assessed for trigger specificity, release reproducibility, local toxicity, cumulative exposure, and scalability.

### Delivery-route optimization

Delivery-route optimization represents the final step in EV engineering because the route of administration directly affects EV biodistribution, CNS exposure, systemic clearance, patient compliance, dosing feasibility, and clinical applicability. The main routes for EV-based AD therapy include intranasal, intravenous, oral, subcutaneous, intramuscular, intrathecal, intracerebroventricular, and intracerebral delivery^[159]^. Intranasal administration is non-invasive, repeatable, and can partially bypass the BBB via the olfactory and trigeminal pathways, making it a promising approach for chronic AD therapy^[160]^. Oral delivery is especially relevant for PELNVs because it supports GBA modulation and long-term patient adherence^[161]^. Subcutaneous or intramuscular administration may support sustained systemic exposure, but their efficiency in CNS delivery remains unclear. Intrathecal administration provides direct access to the CSF and bypasses the BBB, although AD-specific evidence is limited and procedural risks should be carefully considered^[162,163]^. Intracerebroventricular or intracerebral administration provides high CNS exposure but is invasive and unsuitable for repeated routine use^[164]^. The choice of administration route should be matched to the EV source, engineering strategy, cargo type, treatment target, disease stage, and safety requirements. Hierarchical engineering provides a clear framework for developing EV-based AD therapies. Engineering strategies should be evaluated according to the level at which they act, including donor-cell programming, surface targeting, cargo loading, hybrid platform construction, controlled release, and administration-route optimization. Each level has specific advantages as well as distinct limitations and safety considerations.

## SYSTEMATIC COMPARISON OF ADMINISTRATION ROUTES FOR EV-BASED AD THERAPY

Efficient and safe delivery of EV-based therapeutics to the brain remains a key determinant of their translational feasibility in AD therapy. The route of administration significantly affects EV biodistribution, brain exposure, systemic clearance, immune responses, dosing frequency, patient compliance, and long-term safety. Therefore, selection of an appropriate administration route should not only maximize CNS accumulation but also balance invasiveness, scalability, repeatability, and clinical practicality. Intranasal administration has emerged as one of the most attractive delivery routes for EV-based AD therapy^[165]^. Preclinical and early clinical studies are increasingly using intranasal administration to deliver MSC-EVs or engineered EVs because this route may enhance CNS bioavailability while maintaining a favorable safety profile^[166]^. However, intranasal delivery also has several limitations, including mucociliary clearance, enzymatic degradation in the nasal cavity, limited administration volume, interindividual anatomical variability of the nasal cavity, and variability in dose retention. Therefore, dose consistency, formulation, stability, nasal residence time, and long-term local safety should be carefully evaluated before clinical application. A structured comparison of these strategies is presented in Table 4 and Figure 3.

**Figure 3:**
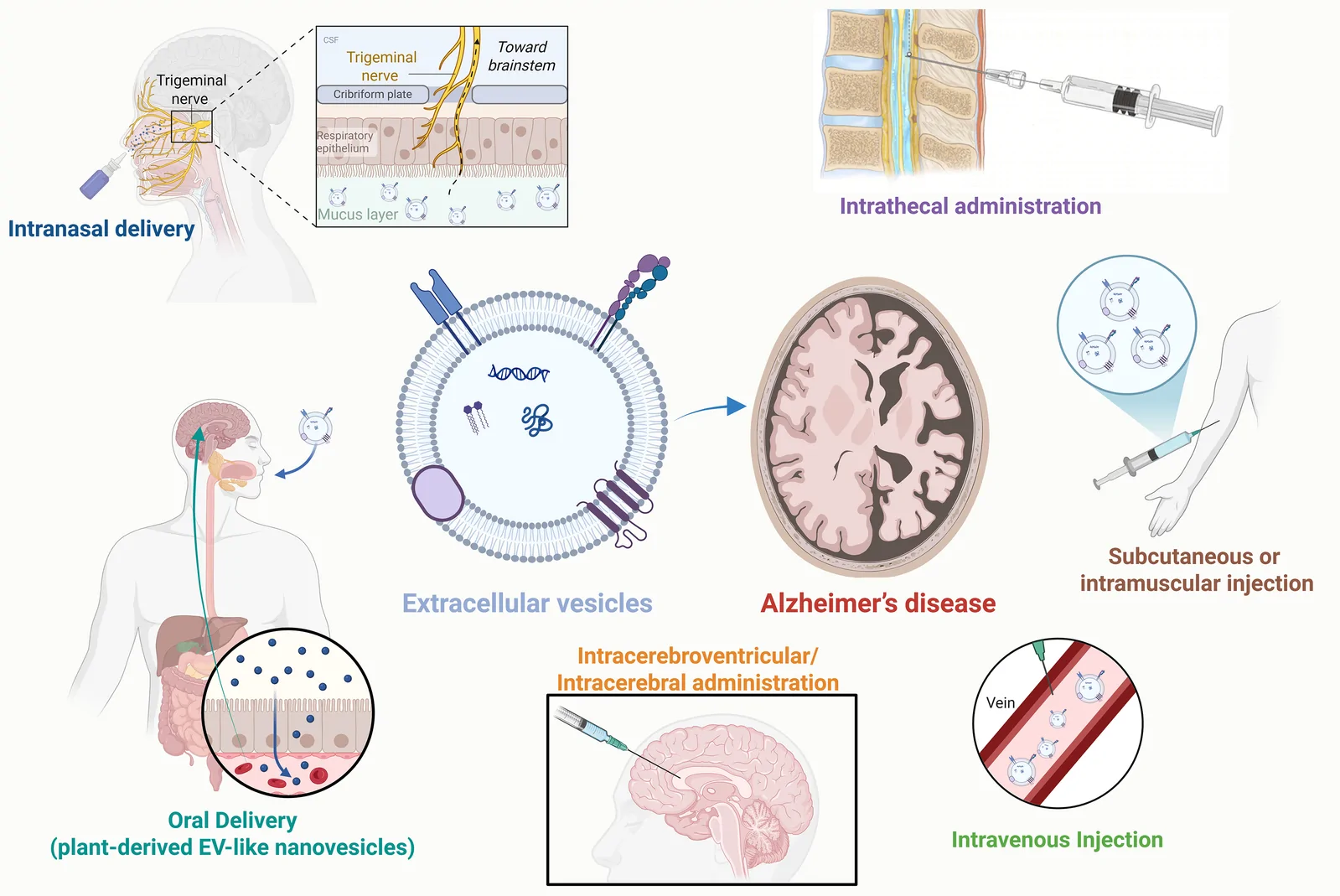
Administration routes of EVs for AD therapy. Created in BioRender. (2026) https://BioRender.com/qjcs5xm. AD: Alzheimer’s disease; CSF: cerebrospinal fluid; EVs: extracellular vesicles.

**Table 4:** Comparison of major administration routes for EV-based AD therapy

**Administration route**	**Main advantages**	**Main limitations**	**Suitable EV types or therapeutic scenarios**	**Ref.**
Intranasal administration	Non-invasive; repeatable; may bypass the BBB through olfactory and trigeminal pathways; reduced systemic exposure	Limited dose volume; mucociliary clearance; variability in nasal absorption; need for formulation optimization	MSC-EVs, engineered EVs, brain-targeted EVs for chronic CNS therapy	[165,166]
Intravenous injection	Clinically familiar; scalable; suitable for systemic delivery; useful for peripheral immune modulation	Rapid clearance by liver, spleen, lung, and kidney; limited brain accumulation; possible off-target effects	Engineered EVs with targeting ligands; EVs designed for systemic immunomodulation	[167,168]
Oral delivery	Convenient; high patient compliance; especially suitable for PELNVs; potential GBA modulation	Gastrointestinal degradation; uncertain absorption; variable bioavailability; unclear CNS delivery mechanism	PELNVs, food-derived EVs, GBA-targeted interventions	[169]
Subcutaneous or intramuscular injection	Convenient for repeated dosing; less invasive than direct CNS injection; may support sustained systemic exposure	CNS delivery efficiency uncertain; local immune reactions; variable lymphatic transport	Long-term systemic immunomodulation; exploratory sustained-release EV strategies	[170]
Intracerebroventricular or intracerebral administration	Direct CNS exposure; high brain delivery efficiency; useful for mechanistic studies	Highly invasive; procedural risks; poor suitability for repeated administration; limited clinical feasibility	Preclinical proof-of-concept studies or severe cases requiring direct CNS delivery	[171]
Intrathecal administration	Direct delivery into CSF; bypasses BBB; lower systemic exposure; less cranially invasive than intracerebroventricular or intracerebral injection	Invasive lumbar puncture; possible infection, bleeding, meningeal irritation, uneven CSF distribution and rapid CSF clearance; AD-specific evidence and optimal dose remain unclear	Exploratory CNS-targeted EV delivery, nucleic acid-loaded EVs, or cases requiring direct CSF exposure; currently mainly supported by non-AD CNS evidence	[163,172-174]

AD: Alzheimer’s disease; BBB: blood-brain barrier; CNS: central nervous system; CSF: cerebrospinal fluid; EVs: extracellular vesicles; GBA: gut-brain axis; MSC-EVs: mesenchymal stem cell-derived extracellular vesicles; PELNVs: plant-derived exosome-like nanovesicles.

Intravenous injection is another widely used route of administration because it is technically straightforward, clinically well established, and suitable for large-scale systemic delivery. This route is particularly useful when EVs are designed to exert both central and peripheral immunomodulatory effects^[167]^. However, intravenously administered EVs are often rapidly cleared by the mononuclear phagocyte system and tend to accumulate in organs such as the liver, spleen, lung, and kidney, resulting in limited brain delivery efficiency^[168]^. Surface engineering strategies, including RVG peptide modification, ligand conjugation, and membrane functionalization, can improve BBB penetration and brain targeting. These modifications may also change the pharmacokinetic characteristics, immunogenicity, and toxicity of EVs. Therefore, intravenous EV therapy requires comprehensive biodistribution analysis, dose optimization, repeated-dose toxicity evaluation, and evaluation of off-target organ accumulation.

Oral administration is particularly suitable for PELNVs. These vesicles have received wide attention because of their high natural abundance, low production cost, good biocompatibility, and suitability for large-scale production^[175]^. Compared with mammalian cell-derived EVs, PELNVs may be more stable in the gastrointestinal environment and can interact with intestinal epithelial cells, immune cells, and gut microbiota^[175,176]^. This characteristic makes oral administration particularly promising for GBA-targeted AD interventions. By regulating intestinal inflammation, microbial composition, metabolic signaling, and systemic immune response, oral PELNVs can indirectly affect CNS inflammation and neurodegenerative processes^[169]^. However, oral EV administration also faces a number of major challenges, including gastrointestinal degradation, variable absorption efficiency, uncertain pharmacokinetic characteristics, inter-batch heterogeneity, and limited understanding of the mechanisms by which these EVs and their cargo reach or influence the brain. Future research should clarify their intestinal uptake mechanism, metabolic fate, bioactive cargo stability, and long-term safety.

Subcutaneous and intramuscular injections can be used as alternative routes for repeated EV administration, especially when continuous systemic exposure or immune modulation is required. Compared with intracerebral injection, these administration routes are less invasive; for long-term treatment, they are also more convenient than repeated intravenous infusion. They may also promote the gradual absorption of EVs through local tissue and lymphatic circulation, thus prolonging the circulation time and reducing the toxicity associated with peak concentration^[170]^. However, whether subcutaneously or intramuscularly administered EVs can achieve sufficient CNS exposure remains uncertain. Local immune response, inflammation at the injection site, degradation by tissue resident phagocytes, and different lymphatic transport pathways may all limit therapeutic efficacy. At present, these administration routes are more suitable for exploratory research or systemic immunomodulatory strategies than for EV therapy directly targeting the brain.

Intracerebroventricular or intracerebral administration can directly deliver EVs to the CSF or brain parenchyma, thus increasing CNS exposure and reducing reliance on BBB transport^[171]^. This method is suitable for mechanistic research and proof-of-concept experiments. Its clinical application in AD is limited by high invasiveness, risks of infection or bleeding, low patient acceptance, and poor suitability for repeated long-term administration. Direct CNS administration can achieve high delivery efficiency, but it is unlikely to become the preferred route for routine EV-based AD therapy.

Intrathecal administration is another potential route for delivering EV-based therapeutics to the CNS. Unlike intravenous administration, intrathecal injection introduces EVs directly into the CSF through the spinal subarachnoid space, bypassing the BBB and reducing systemic exposure^[173]^. Compared with intracerebroventricular or intracerebral injection, this method can avoid direct cranial surgery and may have higher clinical feasibility in specific neurological diseases. Intrathecal administration is especially important for EVs that carry nucleic acids, proteins, or other macromolecular cargo that need to enter the CNS efficiently. Evidence supporting this route in AD remains limited. A recent systematic review of EV-based therapy pointed out that most *in vivo* studies use intravenous administration, followed by intranasal administration and intracerebral administration; intrathecal administration has not yet become the main administration strategy for Alzheimer’s disease-specific EV research^[172]^. The potential value of intrathecal EV administration in AD is currently mainly based on broader CNS disease models and EV-mediated gene delivery research. In these studies, intrathecal delivery of EVs has been used to achieve CNS-directed nucleic acid delivery and has been verified in spinal cord injury models. The results show that EVs can reach the CSF compartment and may produce neuroprotective or regenerative effects^[174,177]^. Intrathecal administration has potential advantages, but it is still an invasive procedure and there are some challenges in clinical translation and application. These risks include procedure-related complications, such as infection, bleeding, meningeal irritation, uneven distribution of CSF, rapid CSF clearance, and limited feasibility for repeated administration. AD-specific studies should compare the effects of intrathecal administration with those of intranasal, intravenous, and intracerebral administration routes, and focus on drug distribution in the CSF, penetration from the CSF into brain parenchyma, dose-response relationship, safety characteristics, and cognitive function changes.

For EV-based AD treatment, there is no universally applicable single administration route. Intranasal delivery is suitable for non-invasive brain-targeted treatment, while intravenous injection is suitable for large-scale systemic administration. Oral administration has shown potential for PELNV-based GBA modulation, while subcutaneous or intramuscular injection may help achieve long-term systemic immunomodulation. Translational studies should compare these routes using standardized pharmacokinetic, biodistribution, safety, and efficacy endpoints. The optimal route depends on EV source, engineering strategy, cargo type, disease stage, treatment frequency, and whether the goal is direct CNS delivery or systemic modulation of AD-related pathology. EV engineering also supports combination therapy. By enhancing brain targeting, cargo loading, and controlled release, engineered EVs can be combined with existing AD treatments, including AChEIs, anti-Aβ antibodies, and non-pharmacological interventions, to achieve multi-target regulation of AD pathology.

## CLINICAL TRANSLATION AND CHALLENGES

EVs, particularly MSC-EVs, are promising candidates for AD therapy owing to their low immunogenicity, potential scalability from standardized donor cell lines, relatively non-invasive administration options, and favorable storage properties. Based on the literature identified from PubMed and ClinicalTrials.gov, with the last search conducted on May 30, 2026, clinical translation of EV-based AD therapies remains at an early stage but shows encouraging potential. Among the limited clinical studies identified, MSC-derived EVs and MSC-derived EV-containing preparations are the main therapeutic modalities reported to date, with intranasal administration explored for its ability to bypass the BBB and enable repeated, non-invasive dosing. The most advanced clinical example is the Phase I/II open-label study at Ruijin Hospital (NCT04388982)^[178]^, in which intranasal ahaMSC-EVs were given to patients with mild-to-moderate AD. The treatment showed a favorable safety profile, with no EV-related adverse events and early signs of cognitive improvement. A later peer-reviewed report supported these findings and provided dose references and feasibility data for future randomized controlled trials. A related single-arm feasibility study in Japan evaluated intranasal administration of MSC secretome containing EVs and soluble factors, rather than purified EVs, in patients with AD^[179]^. Although the study did not use purified EVs, the intervention was well tolerated, improved Hasegawa Dementia Scale-Revised (HDS-R) scores, and showed that self-administration at home was feasible. These findings highlight the broader therapeutic potential of EV-rich biological agents and the need to clarify product definitions and standardization. Early clinical data are encouraging, but EV-based AD therapy still faces major translational challenges, including GMP-compatible large-scale manufacturing, standardized isolation and purification, reproducible batch release standards, dose selection for different administration routes, and disease-related efficacy evaluation. Future clinical trials should adopt a systematic treatment framework to evaluate the impact of EV interventions on cognitive function, Aβ/tau biomarkers, neuroinflammation, mitochondrial dysfunction, synaptic repair, functional outcomes, and long-term safety. Clinical studies of EV-based or EV-containing approaches in AD are summarized in Table 5.

**Table 5:** Clinical trials and clinical studies related to EV-based or EV-containing therapies for AD

**Evidence category**	**Trial/Registry**	**Product category**	**Product (EV type/source)**	**Route and dosing**	**Population**	**Primary endpoints**	**Status/year**	**Notes**	**Ref.**
Registered clinical trial with published clinical report	NCT04388982 (Ruijin Hospital)	Purified EV product	ahaMSC-EVs	Intranasal; multi-dose exploratory schedule	Mild-to-moderate AD	Preliminary safety and feasibility were reported; no EV-related adverse events were observed, with exploratory signals of cognitive improvement	Completed; clinical report published 2023	This is a registered clinical trial of an EV-based therapeutic product. The published clinical report corresponds to this registered trial and provides early clinical evidence for intranasal MSC-EVs in AD.	[178]
Published clinical study of EV-containing secretome	Japan single-arm feasibility study	EV-containing secretome, not purified EV product	MSC secretome/conditioned medium containing EVs and soluble factors	Intranasal; 3 vials/week for 8 weeks	AD	Reported feasibility, safety, and improvement in HDS-R score	Published 2024	This study investigated MSC secretome rather than purified EVs; therefore, it should be interpreted as supportive but indirect evidence for EV-containing therapeutic preparations	[179]

AD: Alzheimer’s disease; ahaMSC-EVs: allogeneic human adipose mesenchymal stem cell-derived extracellular vesicles; EVs: extracellular vesicles; HDS-R: Hasegawa Dementia Scale-Revised; MSCs: mesenchymal stem cells; NCT: ClinicalTrials.gov identifier.

## SAFETY AND TOXICOLOGICAL CONSIDERATIONS FOR EV-BASED AD THERAPY

Although EV-based therapies have demonstrated considerable potential in AD intervention, their clinical application necessitates the development of a specialized, systematic, and mechanism-informed safety assessment framework. Unlike traditional small-molecule drugs, EVs are biologically active, heterogeneous nanoscale particles, and their safety depends not only on the therapeutic cargo but also on multiple additional factors. These include the origin and physiological status of donor cells, culture and conditioning protocols, methods of EV isolation and purification, surface composition, engineering strategy, administration route, dosing frequency, disease stage, and batch-to-batch production consistency.

The origin of donor cells and the composition of EV cargo are fundamental determinants of EV safety. EVs derived from healthy or therapeutically primed MSCs, NSCs, astrocytes, or anti-inflammatory microglia may carry neuroprotective proteins, regulatory miRNAs, antioxidant molecules, and immunomodulatory signals^[180,181]^. In contrast, EVs released from stressed neurons, disease-related glial cells, inflammatory donor cell cultures, or poorly controlled production systems may contain pathogenic Aβ species, phosphorylated tau proteins, pro-inflammatory cytokines, complement-related proteins, or dysregulated miRNAs^[180,182]^. Such harmful cargo may promote abnormal protein aggregation, glial cell activation, synaptic impairment, and neuronal dysfunction. Therefore, EV preparations intended for AD therapy require comprehensive molecular characterization before clinical application. This should include assessment of Aβ/tau burden, inflammatory mediators, miRNA signatures, protein contaminants, and disease-related molecular patterns.

Immunogenicity and abnormal immune regulation should also be systematically evaluated. EVs, particularly MSC-derived EVs, are generally considered to exhibit relatively low immunogenicity^[183]^. However, low immunogenicity does not imply the absence of immune-related risks. This problem is particularly relevant in AD because neuroinflammation is a central driver of disease progression^[60]^. EVs engineered to suppress neuroinflammation may provide therapeutic benefits; nevertheless, excessive or non-specific immunomodulation may disrupt the surveillance function of microglia, the supportive and homeostatic roles of astrocytes, peripheral immune balance, or host defense mechanisms. Therefore, safety evaluation should include analyses of cytokine release, complement activation, anti-EV antibody formation, microglial activation state, astrocytic reactivity, and systemic immune profiles.

Biodistribution, pharmacokinetics, and off-target accumulation should be evaluated in a route-specific manner. Intravenous administration of EVs is clinically convenient and scalable; however, intravenously administered EVs are often rapidly taken up by the liver, spleen, lung, kidney, and mononuclear phagocyte system, which may reduce delivery efficiency and increase peripheral tissue exposure^[168]^. Intranasal administration can enhance direct transport from the nasal cavity to the brain and reduce systemic exposure, but dose retention, mucociliary clearance, nasal epithelial safety, and interindividual variability remain important considerations. Oral delivery, particularly for PELNVs, may be suitable for modulating the GBA^[161]^. However, the gastrointestinal degradation, absorption efficiency, metabolic fate, and CNS-related effects of orally administered EVs remain incompletely understood. Although direct CNS administration, including intrathecal, intracerebroventricular, or intracerebral delivery, can improve EV distribution in the CSF or brain parenchyma, it also introduces procedure-related risks, such as infection, hemorrhage, meningeal irritation, local tissue damage, and limited feasibility of long-term repeated administration. Therefore, route-specific studies of biodistribution, clearance kinetics, organ accumulation, and tolerability are essential for optimizing EV-based AD therapy.

Mechanism-specific CNS safety risks are closely related to the pathological processes targeted by EV-based interventions. EVs designed to modulate Aβ and tau pathology should be carefully screened to prevent the unintended transfer of pathogenic Aβ oligomers, tau seeds, or other aggregation-prone proteins. EVs developed to regulate neuroinflammation should preserve the physiological functions of microglia and astrocytes, including synaptic pruning, neurotransmitter recycling, BBB maintenance, and immune surveillance. EVs targeting oxidative stress or mitochondrial dysfunction should avoid excessive suppression of physiological ROS signaling, disruption of mitochondrial turnover, or impairment of neuronal energy metabolism. EVs intended to enhance synaptic plasticity, remyelination, cholinergic neuroprotection, or neurogenesis should be evaluated for potential effects on neural circuit stability, excitation-inhibition balance, ectopic neurogenesis, seizure threshold, and maladaptive plasticity. These mechanism-specific risks should be comprehensively assessed using behavioral testing, electrophysiology, neuropathology, mitochondrial function assays, neuroinflammatory markers, BBB integrity evaluation, and long-term cognitive monitoring.

The safety of engineered EVs should be evaluated separately from that of native EVs. Surface modification, RVG peptide display, ligand conjugation, membrane hybridization, cargo loading, and stimuli-responsive release systems may improve brain targeting and therapeutic efficacy, but may also alter EV recognition, biodistribution, clearance kinetics, immunogenicity, tissue retention, and toxicity profiles. Cargo-loaded EVs carrying miRNAs, siRNAs, mRNAs, enzymes, neurotrophic factors, mitochondrial regulators, or small molecules may induce off-target gene regulation, innate immune activation, abnormal intracellular signaling, or dose-dependent toxicity. Stimuli-responsive EV systems, such as pH-responsive, magnetic field-responsive, or light-responsive platforms, can provide spatiotemporal control over cargo release, but they may also introduce additional variables related to trigger specificity, local tissue compatibility, release repeatability, and cumulative exposure. Therefore, engineered EVs should be compared with unmodified EVs in terms of physicochemical properties, targeting efficiency, cargo-release capacity, therapeutic potency, biodistribution characteristics, immunogenicity, and repeated-dose safety.

Manufacturing quality and batch-to-batch consistency are key components of toxicological control. The properties of engineered EV preparations may vary depending on donor-cell source, oxygen tension, inflammatory priming conditions, isolation methods, purification strategies, storage conditions, and freeze-thaw cycles. Insufficiently purified or incompletely characterized EV preparations may contain protein aggregates, lipoproteins, nucleic acid contaminants, endotoxins, residual transfection reagents, culture medium components, or non-EV particles. These contaminants may lead to inconsistent efficacy or unexpected toxic reactions^[124]^. Therefore, clinical-grade EV products should include preset quality control standards, including particle size distribution, particle concentration, morphology, EV-enriched marker spectrum, purity, sterility, mycoplasma-negative status, endotoxin level, residual DNA/protein content, cargo identity, potency, and stability profiles^[184]^. For AD therapy, potency should be linked to disease-related functions, such as Aβ clearance, tau protein regulation, neuroinflammatory suppression, mitochondrial protection, synaptic maintenance, and neuronal survival.

Long-term and repeated-dose safety is especially important for AD because it is a chronic, progressive disease. Short-term efficacy studies may miss cumulative neurotoxicity, chronic immune dysregulation, persistent organ accumulation, delayed glial responses, or long-term effects on neural circuit function. Future research should include repeated-dose toxicity testing, dose escalation analysis, recovery assessments, long-term behavioral assessments, organ histopathology examination, CNS biodistribution studies, cognitive follow-up, and biomarker monitoring. The safety assessment of EV-based interventions in AD should go beyond the scope of general toxicity testing and adopt a structured mechanism-based framework instead. This framework should take into account factors such as donor-cell origin, cargo composition, immunogenicity, biodistribution, functional safety of the CNS, engineering-related risks, manufacturing quality, and long-term repeated dosing to define the therapeutic window of EV-based AD therapy.

## DISCUSSION

Although EV-based therapy has shown important advantages for CNS drug delivery, its clinical translation in AD still faces toxicological and safety challenges. Because EVs constitute a biologically active and highly heterogeneous therapeutic category, their safety considerations extend beyond the traditional evaluation frameworks used for small-molecule drugs or conventional biologics, requiring comprehensive and mechanism-based toxicological assessment.

Beyond their therapeutic role, EVs are also emerging as promising non-invasive diagnostic tools and companion biomarkers. EVs can be isolated from a variety of biological fluids, including blood, CSF, urine, and saliva. Importantly, their lipid bilayer structure protects encapsulated proteins, nucleic acids, lipids, and metabolites from enzymatic degradation, thereby preserving disease-relevant molecular information. In the context of AD, neuron-derived or brain-enriched EVs may carry pathogenic and disease-associated molecules, including Aβ species, total tau, phosphorylated tau, inflammatory mediators, synaptic proteins, and specific miRNA signatures. This EV-associated cargo may provide a disease-relevant reflection of CNS pathological changes and may help identify patients at early or prodromal stages of AD.

From a translational perspective, EV-based biomarkers may provide important support for the clinical development and implementation of EV-based therapies. First, EV-associated Aβ, tau, and miRNA profiles may facilitate patient stratification for EV-based interventions, anti-Aβ antibody therapy, or rational combination strategies. Second, longitudinal profiling of EV cargo may allow dynamic monitoring of therapeutic responses, including amyloid clearance, attenuation of neuroinflammation, synaptic repair, and mitochondrial protection. Third, EV biomarkers can be used as an auxiliary diagnostic tool to guide the choice of personalized treatment plans, optimize the timing of treatment, and support precision medicine approaches in AD. Before EV-based diagnostic methods can be transformed into routine clinical practice, several key challenges must be addressed, including standardized EV isolation and characterization procedures to reliably enrich neuron-derived or brain-derived EV subpopulations, validated analytical platforms, and large-scale longitudinal clinical studies.

EV-based therapies for AD can be developed as independent biological intervention methods and can also be incorporated into rational combination strategies. Combining EVs with AChEIs, anti-Aβ antibodies, or lifestyle interventions may achieve broader therapeutic coverage within the complex pathological network of AD. Such combinations may simultaneously provide symptomatic relief, amyloid-targeted clearance, neuroinflammation regulation, synaptic repair, mitochondrial protection, and systemic homeostatic modulation.

## CONCLUSION

EV-based therapeutic technologies have emerged as a multifunctional and biologically adaptable platform for AD therapy, with advantages in brain-targeted delivery, cargo versatility, and biocompatibility. Advances in surface engineering, cargo loading, stimuli-responsive release, and delivery-route optimization have expanded the therapeutic potential of EVs and enabled targeted modulation of Aβ pathology, tau abnormalities, mitochondrial dysfunction, and neuroinflammation in preclinical models. Early clinical studies, especially those using intranasal MSC-EVs, have provided encouraging evidence of feasibility and short-term safety. However, EV-based AD therapies still face major safety and toxicological challenges during clinical translation. Product development should prioritize comprehensive toxicological evaluation, including acute and chronic toxicity, immunogenicity, off-target accumulation, biodistribution, and risks related to repeated administration in chronic neurodegenerative diseases. The full therapeutic value of EVs will depend on the integration of advanced engineering with robust translational frameworks, including GMP-compliant manufacturing, strict quality control, standardized release criteria, and comprehensive safety profiling. Integrating these elements, together with well-designed multicenter clinical trials, will be essential for moving EV-based therapeutics from experimental research toward approved clinical use and for providing safe, effective, and scalable treatment options for patients with AD.

## DECLARATIONS

### Copyright

© The Author(s) 2026.
